# The Effect of Exercise on Cardiovascular Autonomic Nervous Function in Patients with Diabetes: A Systematic Review

**DOI:** 10.3390/healthcare11192668

**Published:** 2023-10-01

**Authors:** Hidetaka Hamasaki

**Affiliations:** Hamasaki Clinic, 2-21-4 Nishida, Kagoshima 890-0046, Japan; h-hamasaki@umin.ac.jp; Tel.: +81-099-250-3535; Fax: +81-099-250-1470

**Keywords:** exercise, diabetes mellitus, cardiovascular autonomic nervous function, heart rate variability, baroreflex sensitivity, diabetic neuropathy, randomized controlled trial

## Abstract

Background: Diabetic neuropathy, including autonomic neuropathy, is a severe complication in patients with poorly controlled diabetes. Specifically, cardiovascular autonomic neuropathy (CAN) plays a significant prognostic role in cardiovascular morbidity and mortality. Exercise, an essential component of diabetes treatment, may have a therapeutic effect on patients with diabetes complicated by CAN. However, it remains unclear whether exercise has a therapeutic or protective effect in diabetes patients with CAN. Methods: The author conducted a systematic search of PubMed/MEDLINE, Embase, and The Cochrane Library, resulting in the identification of eight eligible randomized controlled trials for this review. Results: Exercise, including aerobic exercise combined with resistance training (RT), high-intensity interval training, and progressive RT, has shown a beneficial effect on cardiac autonomic function (CAF) in patients with type 2 diabetes, as measured by heart rate variability, heart rate recovery, and baroreflex sensitivity. However, most studies had low quality. Moreover, there were no relevant studies examining the effect of exercise on CAF in older patients, patients with poorly controlled diabetes, and patients with type 1 diabetes. Conclusions: Exercise has the potential to manage patients with CAN by balancing sympathetic and parasympathetic nervous system functions; however, further studies are warranted in the future.

## 1. Introduction

The autonomic nervous system (ANS) intricately maintains homeostasis throughout the entire human body through a network of neural connections that regulate the function of internal organs and blood vessels. Autonomic function is regulated by the interplay between sympathetic and parasympathetic nervous functions, commonly associated with controlling the “fight or flight” and “rest and digest” responses [1].

Patients with poorly controlled diabetes, whether type 1 diabetes (T1D) or type 2 diabetes (T2D), can develop microvascular complications such as neuropathy, retinopathy, and nephropathy. Diabetic peripheral neuropathy (DPN) is a serious complication clinically presented as diabetic foot and neuropathic pain. Approximately 50% of patients with diabetes suffer from diabetic sensorimotor peripheral neuropathy at some point in their lifetime [2]. Autonomic neuropathy also manifests with clinical symptoms including hypoglycemia unawareness, orthostatic hypotension, dyshidrosis, constipation, diarrhea, erectile dysfunction, and neurogenic bladder [2]. Specifically, cardiovascular autonomic neuropathy (CAN), one of the severe diabetic complications often under-diagnosed, plays a significant prognostic role in cardiovascular morbidity and mortality [3]. The Toronto Consensus Panel on Diabetic Neuropathy defined CAN as “impairment of cardiovascular autonomic control in patients with established diabetes after excluding other causes” [4,5,6]. Considering that CAN is common in individuals with impaired glucose tolerance [7], it may already be present in patients with diabetes at the time of diagnosis, making early diagnosis and intervention crucial. A recent meta-analysis showed that CAN is associated with an increased risk of future cardiovascular events (relative risk (RR) = 3.16; 95% confidence interval (CI), 2.42 to 4.13) and all-cause mortality (RR = 3.17; 95% CI, 2.11 to 4.78) in patients with T1D and T2D [8]. Furthermore, lower heart rate variability (HRV), a cardiac autonomic function test, was associated with a higher risk of heart failure (hazard ratio (HR) = 1.70 for the lowest vs. highest quartile; 95% CI, 1.14 to 2.54) in patients with T2D. Patients with CAN also exhibited a higher risk of developing heart failure (HR = 2.65; 95% CI, 1.57 to 4.48) [9]. While there is no conclusive evidence for pharmacological treatment for CAN, clinical symptoms such as tachycardia, nocturnal hypertension, and orthostatic hypotension can be managed through lifestyle modifications and/or medication. Additionally, detecting CAN may help clinicians appropriately stratify the risk of diabetic complications, cardiovascular morbidity, and mortality and devise effective strategies for diabetes management in asymptomatic patients [3].

Diabetes patients with autonomic neuropathy are at high risk of severe conditions in their management. For example, hypoglycemia unawareness can have severe and potentially life-threatening outcomes, especially in those who have progressed complications and additional medical conditions [10]. Despite the link between CAN and higher mortality rates, there is currently no successful treatment available to prevent or reverse CAN apart from managing blood glucose levels and glucose variability [10]. The current literature suggests that severe hypoglycemia is independently associated with CAN in patients with diabetes [11,12]. Furthermore, experimental hypoglycemia leads to a reduction in HRV and impairs baroreflex sensitivity [13,14]. These findings highlight the importance of avoiding hypoglycemia in diabetes management to prevent CAN in patients with diabetes.

On the other hand, exercise significantly improves HRV-related parameters in patients with T2D, suggesting that exercise can treat ANS dysfunction [15]. Resistance training (RT) combined with aerobic exercise (AE) has shown a favorable effect on autonomic function in older individuals with chronic diseases [16]. Exercise may have a therapeutic effect on diabetes patients complicated with CAN, although it remains unclear whether exercise has a therapeutic or protective effect in diabetes patients with CAN.

This systematic review aims to summarize the current evidence regarding the effect of exercise on CAN in patients with diabetes and discuss its future perspectives and limitations for better diabetes management.

## 2. Materials and Methods

This systematic review was conducted following PRISMA (Preferred Reporting Items for Systematic Reviews and Meta-Analyses) guidelines [17] (Supplementary File). The protocol for this systematic review was not registered in a database such as PROSPERO before conducting the literature search.

### 2.1. Search Strategy

The author conducted a comprehensive search on PubMed/MEDLINE, Embase, and The Cochrane Library, covering the period from their inception to 20 September 2023. The search utilized Medical Subject Headings (MeSH) terms “exercise” AND “diabetes mellitus” AND “randomized controlled trial”, as well as the search term “autonomic”, as the MeSH term “cardiovascular autonomic neuropathy” was not available. Only English-language articles published in peer-reviewed journals were included in the review.

### 2.2. Inclusion and Exclusion Criteria

The articles had to meet the following criteria: (1) the study design had to be a randomized controlled trial (RCT), (2) the study population had to consist of adults with diabetes, (3) the exercise intervention had to involve structured exercise, and (4) the study had to examine whether there was a change in cardiovascular autonomic function (CAF) following the intervention. The author reported study results not only for CAF-related indices but also for endocrine- and immune-system-related parameters in order to comprehensively describe the results of previous RCTs. Reviews, non-randomized trials, observational studies, case reports, commentaries, editorials, letters, conference papers, and study protocols were excluded. RCTs examining the effects of other exercise modalities such as yoga and breathing exercises were also excluded.

### 2.3. Study Quality Assessment

The revised Cochrane risk-of-bias tool for randomized trials was used to assess the quality of the included studies [18]. Bias is assessed for individual elements in five domains: randomization process, deviations from the intended interventions, missing outcome data, measurement of the outcome, and selection of the reported results. The assessment was categorized into one of three levels: “high risk of bias,” “some concerns,” and “low risk of bias,” based on the risk-of-bias tools [19].

## 3. Results

### 3.1. Study Selection

The systematic literature search yielded a total of 63 articles. Among these, 24 articles were excluded as they did not meet the criteria for being RCTs. The titles and abstracts of the remaining articles were reviewed to determine their relevance. Additionally, 28 articles were excluded based on factors such as article types, study subjects, exercise modalities, and study outcomes. As a result, a total of 11 articles were identified for inclusion in this systematic review. However, the study by Sacre et al. [20] was excluded from the review because the authors stated in their full text that the randomization process had failed. The study by Bellavere et al. [21] was also excluded because, although it was a randomized study comparing the effect of AE and RT, it did not include a control group. Furthermore, the study by Cruz et al. [22] was excluded because it did not examine the change in CAF after the intervention. Ultimately, a total of eight RCTs were included in this systematic review.

**Figure 1:**
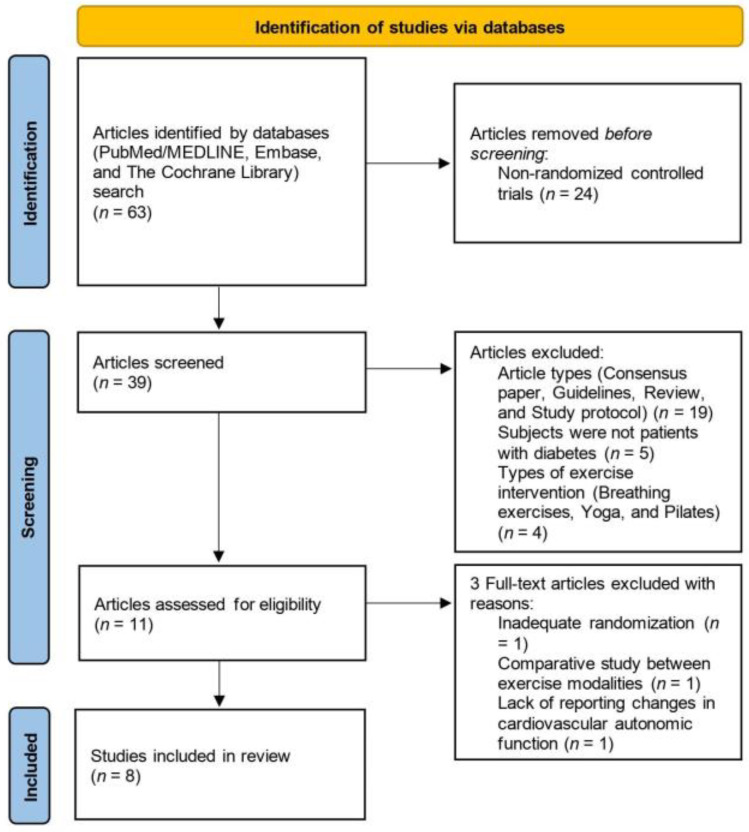
PRISMA flow diagram.

Figure 1 depicts the flow of the systematic search process.

### 3.2. Study Characteristics

To the best of the author’s knowledge, the study conducted by Loimaala [23] represents the first RCT that investigated the impact of exercise on CAF in T2D patients by evaluating baroreflex sensitivity and HRV. Fifty male patients diagnosed with T2D were recruited, with forty-nine patients (exercise group: *n* = 24, control group: *n* = 25) completing the study. Participants in the exercise intervention group were instructed to engage in either walking or jogging, targeting a heart rate level of 65% to 75% of their maximal oxygen consumption (VO_2max_) using a heart rate monitor, twice per week. They also performed progressive RT (eight exercises targeting large muscles in the trunk and upper and lower extremities), consisting of three sets of 10 to 12 repetitions at an intensity of 70% to 80% of their one-repetition maximum (1-RM) twice per week, totaling 104 exercise sessions. The exercise program spanned a duration of 12 months. Baroreflex sensitivity was assessed by measuring the variation in R-R intervals in response to changes in systolic blood pressure. Time-domain HRV was evaluated using the standard deviation of normal R-R intervals (SDNN) and the percentage of adjacent R-R intervals differing by more than 50 ms divided by the total number of R-R intervals (pNN50). Resting heart rate showed a decrease (from 68.8 ± 10 bpm to 65.7 ± 8 bpm), while baroreflex sensitivity showed an increase (from 6.8 ± 2.9 ms/mmHg to 8.6 ± 4.6 ms/mmHg) in the exercise group. In contrast, the control group exhibited an increase in resting heart rate (from 66.3 ± 9 bpm to 68.1 ± 9 bpm) and a decrease in baroreflex sensitivity (from 7.5 ± 3.8 ms/mmHg to 6.4 ± 3.5 ms/mmHg). However, no significant changes in HRV variables were observed. The change in baroreflex sensitivity demonstrated an inverse correlation with the change in HbA1c levels. The authors postulated that the enhancement in baroreflex sensitivity could be attributed to the improvement in endothelial function and blood flow, which are typically impaired in patients with T2D. This improvement may be observed as a result of long-term exercise intervention.

In contrast, Kang et al. [24] reported that exercise based on the recommendations of the American College of Sports Medicine and the American Diabetes Association (i.e., AE should be performed at an intensity of 40–60% of VO_2max_, for a minimum of 150 min per week, on at least 3 days per week, with no more than 2 consecutive days between sessions; RT should be conducted at an intensity of 50–80% of 1-RM, with 8–10 repetitions, involving 5–10 exercises targeting major muscle groups; RT should be performed at least twice a week, on nonconsecutive days [25]) did not have a favorable impact on CAF in patients with T2D. Sixteen female patients diagnosed with T2D were randomly assigned to either the exercise intervention group (*n* = 8) or the control group (*n* = 8). The participants’ HRV, including indicators such as SDNN, root mean square successive differences (rMSSD), low frequency (LF), high frequency (HF), and the LF/HF ratio, was measured using an HRV analyzer in the morning. The exercise intervention consisted of a combined AE and RT program. The participants in the exercise group performed treadmill walking at an intensity of 60% of their heart rate reserve for 30 min, three times per week, over a period of 12 weeks. Following the AE session, participants performed RT for 30 min during which they completed two sets of 8–12 repetitions (60–80% of 1RM) of nine exercises (leg press, leg extension, leg curl, calf raise, curl up, arm curl, shoulder press, lateral pull-down, and chest press) using weight machines. After the 12-week AE and RT program, improvements were observed in body composition, muscle strength, VO_2max_, glycemic control, blood pressure, and insulin resistance. However, no significant change in HRV was observed.

Liu et al. [26] conducted a study that investigated two aspects: (1) the association between heart rate recovery (HRR) after exercise and cardiovascular risk factors and (2) the effect of a combined moderate-intensity AE and RT on HRR in patients with T2D. Abnormal HRR was defined as a decrease in heart rate of less than 18 bpm from the maximum heart rate to one minute after exercise. The relationship between abnormal HRR and risk factors for cardiovascular disease was examined before the exercise intervention. Logistic regression analyses showed that abnormal HRR was positively associated with fasting blood glucose levels (odds ratio (OR) = 1.9, 95% CI, 1.112 to 3.247), HbA1c levels (OR = 1.74, 95% CI, 1.062 to 2.851), low-density lipoprotein cholesterol levels (OR = 2.325, 95% CI, 1.157 to 4.672), resting heart rate (OR = 2.43, 95% CI, 1.374 to 4.296), and maximum heart rate (OR = 0.595, 95% CI, 0.4 to 0.883). Forty-two T2D patients with abnormal HRR were randomly assigned to either the exercise intervention group (*n* = 22) or the conventional therapy control group (diet therapy plus metformin). The exercise intervention consisted of a supervised exercise training phase (at least three sessions) and a home-based individual exercise phase. Participants engaged in AE for 30 min per session, three times per week, for 12 weeks during the study period. The intensity of AE was set at 40–60% of each participant’s VO_2max_. Participants also performed RT (moderate-intensity, 50–60% of the 1-RM) two to three times per week using elastic bands without the Valsalva maneuver. Compared to the control group, the exercise intervention group showed significant reductions in blood total cholesterol levels, low-density lipoprotein cholesterol levels, and maximum heart rate (from 142.45 ± 13.98 bpm to 135 ± 11.17 bpm). Although HRR increased and resting heart rate decreased in both the control and intervention groups, the changes in HRR (from 12.9 ± 5.71 bpm to 15.75 ± 6.3 bpm vs. from 14 ± 3.47 bpm to 19.77 ± 5.07 bpm) and resting heart rate (from 87.9 ± 7.29 bpm to 82.2 ± 6.16 bpm vs. from 86.59 ± 11.81 bpm to 77.95 ± 5.32 bpm) were greater in the intervention group compared to the control group. Furthermore, maximum metabolic equivalents significantly increased with the exercise intervention compared to conventional therapy alone. HRR reflects the dynamic balance between sympathetic and parasympathetic nervous system functions, providing valuable insights into the evaluation of autonomic dysfunction [27]. The findings of this study suggest that exercise improves autonomic function in patients with T2D.

Cassidy et al. [28] investigated the impact of high-intensity interval training (HIIT) on glycemic control and CAF in patients with T2D using a commercially available non-invasive device to measure hemodynamics and autonomic function. A total of 28 participants were initially assigned to either the exercise intervention group (*n* = 14) or the control group (*n* = 14); however, three participants dropped out from each group. It remains unclear whether the authors conducted intention to treat (ITT) analysis in this study. The HIIT group participated in three exercise sessions per week on nonconsecutive days for a duration of 12 weeks. Each exercise session consisted of a 5 min warm-up followed by high-intensity cycling exercise with a pedal cadence exceeding 80 revolutions per minute, reaching a rating of perceived exertion (RPE) of 16–17 or higher, and then a 3 min recovery period. Five intervals were performed in each exercise session. The duration of HIIT started at 2 min in the first week and increased by 10 s each week. After the 12-week exercise program, the intervention group exhibited a reduction in HbA1c levels by 0.26%, compared to 0.18% in the control group, with a significant difference between the groups. The high-frequency component of systolic blood pressure variability also decreased from 19 ± 4% to 15 ± 3% (a change of 21%) in the HIIT group, while no change was observed in the control group. However, there were no significant changes observed in HRV, including the R-R interval and the LF/HF ratio, or in baroreflex receptor sensitivity. In addition, baseline HbA1c levels were inversely correlated with baroreflex receptor sensitivity. The authors concluded that the effect of HIIT on CAF was limited since it was not associated with improvements in CAF-related indices.

Su et al. [29] examined the effects of AE combined with RT on HRV and serum inflammatory markers in elderly women with T2D. A total of 30 patients aged over 56 years were recruited, and 27 patients (exercise group: *n* = 14, control group: *n* = 13) completed the study. Participants in the exercise intervention group performed AE combined with RT for 60 min per session, three times per week, over a period of 12 weeks. AE involved treadmill walking at an intensity of 65–70% of the maximum heart rate, while RT was performed using dumbbells, an elastic belt, and other training techniques. The intensity of RT was set at 70–85% of 1-RM. After the 12-week exercise program, the intervention group showed significant increases in SDNN (from 108.93 ± 6.66 ms to 127.71 ± 3.36 ms), rMSSD (from 38.54 ± 4.31 ms to 48.14 ± 3.30 ms), and HF (from 4.26 ± 0.52 ms^2^ to 4.81 ± 0.35 ms^2^) compared to the control group. Furthermore, the intervention group exhibited a significantly lower LF/HF ratio (from 0.98 ± 0.16 to 0.81 ± 0.13) and resting heart rate (from 75.14 ± 4.77 bpm to 72.00 ± 4.45 bpm) compared to the control group. The exercise intervention also resulted in significant reductions in C-reactive protein (CRP), interleukin (IL)-6, and tumor necrosis factor (TNF)-α levels compared to the control group.

In a recent study by Bhati et al. [30], the effect of RT on CAF was comprehensively evaluated using various cardiovascular autonomic reflex tests based on Ewing’s criteria [31] in T2D patients with CAN. Initially, 56 patients participated in the study, but four participants from each group dropped out of the study. The authors explicitly stated that all patient data were evaluated using ITT analysis. Participants in the exercise intervention group engaged in progressive moderate-intensity RT (65–75% of 1-RM) three times per week for 12 weeks. Each session included two to three sets of 8–12 repetitions, lasting approximately 60 min. The RT program comprised ten exercises: leg press, knee extension, standing leg curls, abdominal curls, lat pull-downs, seated rows, butterfly, bicep curls, overhead tricep extensions, and bench press. The 12-week RT program improved various variables related to parasympathetic cardiac control. These included the ratio of the average of the longest R-R interval during expiration to the shortest R-R interval during inspiration in the deep breathing test, heart rate change during the deep breathing test, and the Valsalva ratio. Additionally, time-domain HRV variables at rest, such as SDNN (from 30.6 ± 18.84 ms to 34.5 ± 17.88 ms), rMSSD (from 26.4 ± 14.60 ms to 35.7 ± 13.85 ms), pNN50 (from 1.73 ± 1.35% to 2.91 ± 2.03%), and the standard deviation of successive differences (SDSD) (from 23.0 ± 15.85 ms to 31.5 ± 17.08 ms), showed either a significant group effect, a group-by-time interaction effect, or both. In the frequency domain, all HRV variables (LF, HF, and the LF/HF ratio) demonstrated a significant group-by-time interaction effect, indicating a favorable effect of RT on resting HRV. Notably, significant group-by-time interaction effects were also observed in post-exercise HRV, including SDNN, rMSSD, pNN50, SDSD, HF power, and HRR at 30 s and 1 min after exercise, except for LF and the LF/HF ratio. Both α-LF (sympathetic-nervous-system-mediated baroreflex sensitivity) and α-HF (parasympathetic-nervous-system-mediated baroreflex sensitivity) showed significant interaction effects after the 12-week RT, suggesting that RT significantly influences baroreflex sensitivity gain. Moreover, RT may play an anti-inflammatory role in vasculature. Serum IL-6 and IL-18 levels significantly decreased by 12.6% and 12%, respectively, following RT. In addition, endothelial nitric oxide synthase (eNOS) levels significantly increased by 32.6% after RT, while other biomarkers, such as angiotensin II, did not exhibit significant changes. The authors argued that the significant improvements in baroreflex sensitivity and eNOS concentrations were a result of enhanced cardiac parasympathetic modulation and reduced cardiac sympathetic modulation through the baroreflex–NO axis. According to the current literature, there is a correlation between skeletal muscle function and the balance between the sympathetic and parasympathetic nervous systems. Sarcopenic patients with heart failure had higher muscle sympathetic nerve activity and showed a lower change in HRR compared to non-sarcopenic patients [32].

Michou et al. [33] investigated the effects of home-based AE combined with RT on CAN-related parameters and metabolic profiles in diabetic kidney disease patients undergoing hemodialysis. Forty-one patients were randomly assigned to the exercise intervention group (*n* = 21) and the control group (*n* = 20). However, the number of patients completing the study was 15 in the intervention group and 13 in the control group, respectively. The exercise program consisted of AE and RT, with three sessions per week for 6 months. Patients in the intervention group performed AE activities such as walking or cycling at an intensity of 50–70% of predicted VO_2peak_ and RT exercises for the upper and lower extremities using their body weight, rubber bands, balls, or dumbbells (two sets, 8–10 repetitions) on non-dialysis days. This 6-month home-based exercise program was individualized by monitoring the patient’s adherence to the program and their exercise progress. On the other hand, patients in the control group received usual care and were asked not to participate in any structured exercise. The 6-month exercise program significantly improved SDNN (from 95.46 ± 15.02 ms to 126.40 ± 21.95 ms), rMSSD (from 55.06 ± 25.92 ms to 70.53 ± 23.73 ms), pNN50 (from 8.66 ± 7.20% to 12.20 ± 11.16%), and LF (from 155.53 ± 53.91 ms^2^ to 108.28 ± 48.04 ms^2^). Compared to the control group, the exercise program increased SDNN by 34.3%, rMSSD by 21.5%, and pNN50 by 51.7% and decreased LF by 29.7%. Furthermore, the exercise program increased high-density lipoprotein cholesterol levels and decreased HbA1c levels. In addition, the exercise program improved cardiorespiratory fitness, with a 9.3% increase in VO_2peak_ for patients in the intervention group compared to the control group, and VO_2peak_ was positively correlated with SDNN and HF at the end of the study. This study is noteworthy because it demonstrates the beneficial effect of exercise on CAN-related parameters in patients with advanced diabetic kidney disease. The dropout rate was high (approximately 30%); however, there were no severe adverse events, including cardiovascular complications, in patients who completed the exercise program.

The same researchers also examined the effect of home-based exercise on CAN-related parameters in kidney transplant recipients with T2D [34]. Thirty patients with CAN were enrolled in this study, and five patients dropped out. A total of 25 patients (13 patients in the exercise intervention group and 12 patients in the control group, respectively) completed the study. The exercise program was similar to the previous study [33]. The 6-month exercise program significantly improved total frequency power (from 2408.4 ± 1728.3 ms^2^ to 2549.5 ± 1827.3 ms^2^), SDNN (from 98.6 ± 8.7 ms to 127.7 ± 10.7 ms), the standard deviation of the 5 min average NN intervals (SDANN) (from 105.5 ± 38.5 ms to 139.1 ± 20.1 ms), rMSSD (from 38.7 ± 23.2 ms to 51.9 ± 28.0 ms), pNN50 (from 6.4 ± 4.8% to 8.0 ± 5.0%), LF (from 292.8 ± 213.7 ms^2^ to 254.1 ± 199.7 ms^2^), the very low frequency (VLF) (from 2844.5 ± 1753.5 ms^2^ to 2088.4 ± 1401.2 ms^2^), and HF (from 706.4 ± 446.2 ms^2^ to 852.3 ± 502.1 ms^2^). Compared to the control group, the exercise program increased SDNN by 30.3%, SDANN by 32.9%, rMSSD by 32.0%, pNN50 by 29.0%, and HF by 21.6% and decreased LF by 13.2%, VLF by 26.2%, and the LF/HF ratio by 24.0%. The cardiorespiratory fitness and functional capacity of patients in the intervention group also improved. At the end of the study, VO_2peak_ was positively correlated with SDNN. The findings of this study are important, as the study participants were diagnosed with CAN. However, the authors did not describe the process of the diagnosis and the severity of CAN in them.

Table 1 summarizes the characteristics of the included studies.

### 3.3. Study Quality

**Figure 2:**
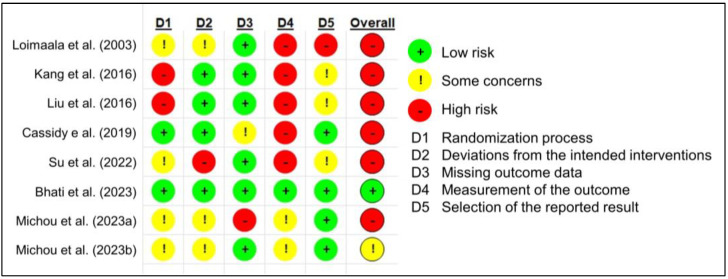
Risk of bias in the included studies [23,24,26,28,29,30,33,34].

The overall risk of bias was “high” or “some concerns” except for the study by Bhati et al. [25] (Figure 2).

Most studies involved a high risk of bias in measuring the outcomes because, except for the study by Bhati et al. [30], the reviewed RCTs did not seem to conduct blinding. The author could not identify study protocols of the study by Loimaala et al. [23], Kang et al. [24], Liu et al. [26], Su et al. [29], and Michou et al. [33,34] in a reliable registry such as ClinicalTrials.gov [35]. There were also some concerns or high risk of bias in the randomization process because the differences in the characteristics of participants, such as age, gender, BMI, glycemic control, and variables related to CAF were not statistically analyzed in the RCTs except for the study by Cassidy e al. [28], Bhati et al. [30], and Michou et al. [33,34]. The dropout rate was low in most studies; however, it was relatively high in the study by Cassidy et al. (21.4%) [28] and Michou et al. (31.7%) [33]. The sample size varied from 16 to 56, which is relatively small. Sample size calculation is one of the most important steps in designing an RCT. Sufficient sample size to detect a statistical difference between the intervention group and the control group depended on the RCT design [36]; thus, the study protocol that explains how to design and estimate sample size is essential to conducting high-quality RCTs.

## 4. Discussion

**Figure 3:**
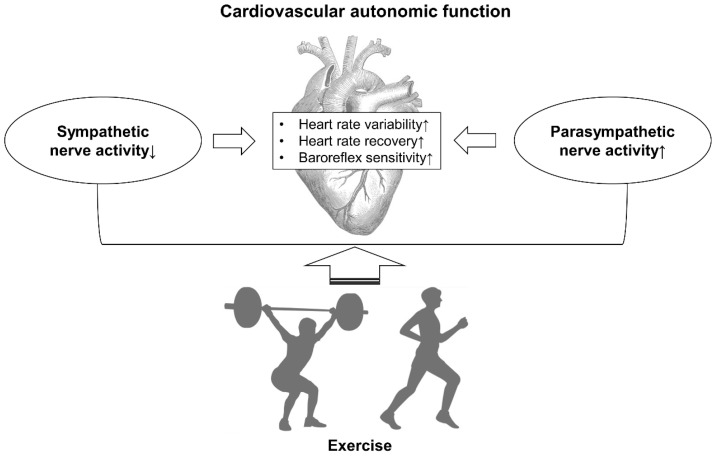
Summary figure describing the main results of this study. ↑ = increase and ↓ = decrease.

This systematic review demonstrates the beneficial effects of exercise interventions on CAF in patients with T2D, as evaluated by HRV, HRR, and baroreflex sensitivity (Figure 3). Spectral analysis of HRV does not directly reflect nerve activity but rather the sympathetic and parasympathetic modulation of the sinoatrial node. However, the effects of regular chronic exercise on CAF-related indices suggest that exercise has an impact on the balance between sympathetic and parasympathetic nervous activity.

The duration of the included studies was 12 weeks, except for the studies by Loimaala et al. [23] and Michou et al. [33,34], and six out of eight studies used AE combined with RT, which aligns with exercise therapy recommendations for diabetes patients. These factors indicate a relatively low heterogeneity in the intervention methods among the studies. While it remains unclear which type of exercise, whether aerobic or anaerobic (e.g., RT and HIIT), is more effective in improving CAF in diabetes patients, exercise has a high likelihood of significantly and favorably impacting CAF in patients with T2D.

The potential mechanisms underlying the effect of exercise on the ANS can be considered as follows: Regular chronic exercise improves sympatho-vagal balance by increasing parasympathetic nervous system activity and decreasing sympathetic nervous activity in patients with T2D [37,38,39]. Three months of regular exercise leads to reductions in blood levels of norepinephrine and N-terminal pro-brain natriuretic peptide. Among these changes, the decrease in norepinephrine levels emerges as the most significant predictor of cardiac mortality in heart failure patients [40]. Moreover, physical exercise restores central sympathetic outflow in rats. Patel et al. demonstrated in their study that exercise exerts a beneficial influence on the inhibitory and excitatory pathways of sympathetic nerve activity within the brain [41]. This effect is mediated by elevated nitric oxide (NO) levels in the paraventricular nucleus, promoting the inhibition of sympathetic nerve activity, as well as reduced levels of central angiotensin II, which contribute to the activation of sympathetic pathways in different brain regions. Nitric oxide plays a pivotal role in modulating cardiac vagal activity. It has been observed that NO concentrations increase in both T1D and T2D patients [42], indicating reduced NO bioavailability in diabetes-induced vascular and endothelial dysfunction [43]. Exercise, particularly AE and combined exercise, has been shown to enhance endothelial function in patients with T2D [44], which could potentially enhance NO bioavailability, thus inhibiting sympathetic nerve activity and enhancing parasympathetic nerve activity. Furthermore, the increase in blood volume resulting from exercise can enhance cardiac vagal modulation by activating the baroreflex [45]. Shimojo et al. [46] reported that moderate-intensity acute exercise resulted in a decrease in serum TNF levels in mice, mediated by subdiaphragmatic vagus nerve activity. Moreover, the study found an increase in anti-inflammatory cytokines, specifically transforming growth factor-β1 and IL-10. Considering the findings of the studies by Su et al. [29] and Bhati et al. [30], there could be an interaction between CAF and inflammation. However, the underlying mechanism of the effect of exercise on ANS function, particularly CAF, is not fully elucidated. Further research is needed in the future.

ANS dysfunction significantly affects the daily lives of patients with diabetes. On the other hand, current evidence supports that exercise, including regular AE, HIIT, and even a single exercise session, improves glycemic variability in patients with T2D [47]. Exercise can potentially induce hypoglycemia in patients with diabetes, particularly those with T1D treated with insulin. However, exercise can also contribute to the improvement of CAF through enhanced glycemic variability. This improvement can be achieved by carefully considering and managing factors such as the timing of exercise, the type of exercise, and the balance between medications and dietary intake [48].

This systematic review has a strength in including only RCTs for review. Picard et al. [15] conducted a systematic review and meta-analysis assessing the effect of exercise on HRV in T2D patients; however, they included not only RCTs but also non-randomized controlled trials, before–after studies, and observational studies in their analysis, which limits the quality of quantitative synthesis. However, there are several limitations that need to be addressed. First, since this study was conducted by a single author, there is a possibility of biases in the selection of studies and the assessment of study quality. Therefore, it is recommended that a systematic review with a meta-analysis be conducted in the future, including a sufficient number of RCTs. The Cochrane Handbook for Systematic Reviews of Interventions suggests a minimum of 10 studies per examined covariate in a meta-analysis [49], thus requiring more high-quality RCTs to fully assess the effect of exercise on CAF in patients with diabetes. Second, to date, there are no RCTs investigating the effect of exercise on CAF in patients with T1D. T1D and T2D differ significantly in terms of etiology and patient characteristics, such as age of onset, pancreatic β-cell function [50], and the prevalence of obesity [51,52]. Therefore, future studies should clarify the effect of exercise on CAF in T1D patients as well. Third, Bhati et al. [30] and Michou et al. [34] enrolled T2D patients with CAN; however, the other included studies investigated the effect of exercise on CAF in patients without CAN. Fourth, the severity of diabetes was mild in the study participants (HbA1c 6.4% to 7.18%), except for the study by Bhati et al. [30]. Given that glucose profile and pancreatic β-cell function are independently associated with the prevalence of CAN [53], the effect of exercise on CAN in patients with poor glycemic control or advanced diabetic complications should also be examined. Fifth, the age of study participants was in their 50s or 60s, and the effect of exercise on CAF in other age groups, particularly older people aged 65 or over, remains unknown. Today, the world is facing an aging population and a declining birth rate [54]. Clinical risk factors for the development and severity of CAN or DPN include age, duration of diabetes, glycemic control, and the presence of other microvascular complications [55,56]. Therefore, data regarding the effectiveness and safety of exercise on CAF in older patients with a long duration of diabetes are critical. Lastly, as mentioned repeatedly by the authors of the included studies, well-designed RCTs with larger sample sizes and longer study periods are needed to establish rigorous evidence on the effect of exercise on CAF in patients with diabetes.

Recent studies have contributed new insights to the scientific evidence in the field of CAF in patients with diabetes. For instance, Vágvölgyi et al. [57] reported that physical training at an intensity of 60–80% of maximal heart rate improved CAF, as assessed by Ewing’s five standard cardiovascular reflex tests, which are the gold standards for diagnosing autonomic dysfunction in patients with metabolic syndrome with and without diabetes. By analyzing data from the Preventing Early Renal Loss in Diabetes (PERL) study and the Action to Control Cardiovascular Risk in Diabetes (ACCORD) study, researchers found that CAN was independently associated with an increased risk of ≥40% glomerular filtration rate decline events (HR = 2.60; 95% CI, 1.15 to 5.45 in the PERL cohort and HR = 1.54; 95% CI, 1.28 to 1.84 in the ACCORD cohort) in both T1D and T2D patients. This suggests that CAN could impact the development of kidney complications in patients with diabetes [58]. Furthermore, the time spent in hyperglycemia was higher in diabetes patients with CAN compared to those without CAN [59]. Diabetes patients with CAN should aim to achieve better glycemic control than those without CAN because intensive therapy in the ACCORD study was associated with a reduced risk of a composite of cardiovascular events in T2D patients with low HRV [60]. Exercise can play a vital role in improving both CAF and glycemic control in diabetes patients with CAN. In the future, various research efforts will continue to advance, further elucidating the clinical significance of treating and managing diabetes patients with CAN while enhancing exercise therapy.

## 5. Conclusions

In conclusion, exercise, including AE combined with RT and progressive RT, has demonstrated a beneficial impact on CAF in patients with T2D. The author cannot conclusively assert that exercise directly improves CAN itself in patients with T2D. However, exercise may play a preventive or mitigating role in severe conditions associated with autonomic neuropathy, such as hypoglycemia unawareness.

However, it is important to note that the quality of most RCTs included in this review was low, highlighting the need for more high-quality research in the future. Nevertheless, exercise therapy shows promise in managing CAN by inhibiting sympathetic nerve overactivity and enhancing parasympathetic nerve activity, and thus improving sympathy-vagal balance and vascular function and potentially reducing inflammation related to ANS function. Exercise therapy is an essential component of the treatment for patients with diabetes, and clinicians should encourage high-risk individuals to engage in regular exercise as part of their daily lives.

## Figures and Tables

**Table 1:** Summary of the included studies.

Reference	Country	Study Design	Study Period	Subjects (Baseline Characteristics)	Primary Study Outcomes	Intervention/Control	Results
Loimaala et al. (2003) [23]	Finland	Randomized, controlled, parallel-group trial	12 months	50 male patients with T2D, 1 dropout Intervention group: Age: 53.6 ± 6.2 years, BMI: 29 3 ± 3.7 kg/m^2^, HbA1c: 8.2 ± 2.1% Control group: Age: 54.0 ± 5.0 years, BMI: 29.8 ± 3.6 kg/m^2^, HbA1c: 8.0 ± 1.3%	Baroreflex sensitivity HRV	AE + RT/conventional therapy	Baroreflex sensitivity↑, resting heart rate↓ SDNN→, pNN50→, HF→, LF→, HF/LF ratio→ VO_2max_↑, muscle strength↑ HbA1c↓, systolic blood pressure↓ Extracellular water↑
Kang et al. (2016) [24]	South Korea	Randomized, controlled, parallel-group trial	12 weeks	16 female patients with T2D, no dropouts Intervention group: Age: 56.0 ± 7.4 years, BMI: 23.9 ± 2.9 kg/m^2^, HbA1c: 6.4 ± 0.6% Control group: Age: 57.5 ± 4.6 years, BMI: 225.5 ± 3.1 kg/m^2^, HbA1c: 6.4 ± 0.5%	HRV	AE + RT/unclear	SDNN→, rMSSD→, LF→, HF→, LF/HF ratio→ VO_2max_↑, grip strength↑ Weight↓, waist circumference↓, body fat percentage↓ HbA1c↓, insulin↓, HOMA-IR↓, systolic blood pressure↓, diastolic blood pressure↓
Liu et al. (2016) [26]	China	Randomized, controlled, parallel-group trial	12 weeks	123 patients with T2D in the cross-sectional analysis 42 patients with T2D Intervention group (12 men and 10 women): Age: 52.6 ± 8.1 years, BMI: unclear, HbA1c: 6.50 ± 0.96% Control group (11 men and 9 women): Age: 53.5 ± 10.7 years, BMI: unclear, HbA1c: 6.91 ± 0.62%	HRR	AE + RT/diet therapy + metformin	HRR↑, resting heart rate↓, max heart rate↓ Fasting blood glucose↓, postprandial glucose↓, HbA1c↓ Fasting insulin↓, postprandial insulin↓ Triglycerides↓, low-density lipoprotein cholesterol↓
Cassidy et al. (2019) [28]	United Kingdom	Randomized, controlled, open-label, parallel-group trial	12 weeks	28 patients with T2D, 6 dropouts Intervention group (9 men and 2 women): Age: 60 ± 3 years, BMI: 31.2 ± 1.70 kg/m^2^, HbA1c: 7.13 ± 0.31% Control group (8 men and 3 women): Age: 59 ± 3 years, BMI: 32.0 ± 1.65 kg/m^2^, HbA1c: 7.18 ± 0.17%	HRV Baroreflex sensitivity Blood pressure variability	HIIT/usual care	R-R intervals→, SDNN→, LF→, HF→, LH/FH ratio→ Baroreflex sensitivity→ Systolic blood pressure HF↓ HbA1c↓
Su et al. (2022) [29]	China	Randomized, controlled, parallel-group trial	12 weeks	30 female patients with T2D, 3 dropouts Intervention group (*n* = 14): Age: 64.01 ± 1.98 years, BMI: 24.90 ± 0.67 kg/m^2^, HbA1c: unclear Control group (*n* = 13): Age: 63.61 ± 2.56 years, BMI: 24.90 ± 0.67 kg/m^2^, HbA1c: unclear	HRV Inflammatory markers	AE + RT/regular treatment	SDNN↑, rMSSD↑, HF↑, LF/HF ratio↑, heart rate↓ IL-6↓, CRP↓ Fasting blood glucose↓, 2-h postprandial glucose↓
Bhati et al. (2023) [30]	India	Randomized, controlled, single blinded, parallel-group trial	12 weeks	56 T2D patients with CAN, no dropout Intervention group (15 men and 13 women): Age: 52.8 ± 6.82 years, BMI: 28.4 ± 3.35 kg/m^2^, HbA1c: 8.4 ± 1.52% Control group (17 men and 11 women): Age: 54.0 ± 8.18 years, BMI: 28.0 ± 3.82 kg/m^2^, HbA1c: 8.2 ± 1.73%	Cardiovascular autonomic reflex tests HRV HRR Baroreflex sensitivity	RT/usual care	SDNN↑, rMSSD↑, pNN50↑, SDSD↑, HF↑, LF/HF ratio↑ Fasting blood glucose↓, postprandial glucose↓, HbA1c↓ IL-6↓, IL-18↓, eNOS↑
Michou et al. (2023) [33]	Greece	Randomized, controlled, parallel-group trial	6 months	28 T2D patients with diabetic kidney disease, 13 dropouts Intervention group (10 men and 5 woman): Age: 62.06 ± 6.34 years, BMI: 28.28 ± 6.22 kg/m^2^, HbA1c: 6.85 ± 0.69% Control group (7 men and 6 women): Age: 63.30 ± 8.50 years, BMI: 29.05 ± 5.71 kg/m^2^, HbA1c: 7.01 ± 1.20%	HRV, Heart rate turbulence Metabolic parameters Cardiorespiratory fitness	AE + RT/usual care	SDNN↑, rMSSD↑, pNN50↑, LF↓ High-density lipoprotein cholesterol↑, HbA1c↓ Resting heart rate↓, METs↑, VO_2peak_↑, systolic blood pressure↓
Michou et al. (2023) [34]	Greece	Randomized, controlled, parallel-group trial	6 months	25 T2D patients with CAN, 5 dropouts Intervention group (10 men and 3 women): Age: 54.9 ± 9.9 years, BMI: 24.8 ± 3.9 kg/m^2^, HbA1c: 6.8 ± 0.2% Control group (9 men and 3 women): Age: 54.0 ± 12.7 years, BMI: 25.6 ± 2.0 kg/m^2^, HbA1c: 6.6 ± 1.0%	HRV, Heart rate turbulence Cardiorespiratory fitness Functional capacity	AE + RT/usual care	SDNN↑, SDANN↑, rMSSD↑, pNN50↑, HF↑, TS↑, LF↓, VLF↓, LF/HF ratio↓ Exercise time↑, METs↑, VO_2peak_↑, exercise HR↑, systolic blood pressure↓ 30 s STS↑, upper limb strength↑, lower limb strength↑

↑ = increase, → = no change, and ↓ = decrease. BMI, body mass index; HbA1c, hemoglobin A1c; T2D, type 2 diabetes; HRV, heart rate variability; AE, aerobic exercise; RT, resistance training; SDNN, the standard deviation of normal R-R intervals; pNN50, the number of pairs of adjacent R-R intervals differing by >50 ms in the entire recording divided by the total number of R-R intervals; HF, high frequency; LF, low frequency; VO_2max_, maximal oxygen uptake; rMSSD, the root mean square successive differences; HOMA-IR, homeostasis model assessment-insulin resistance; HRR, heart rate recovery; HIIT, high-intensity interval training, IL, interleukin, CRP, C-reactive protein; CAN, cardiovascular autonomic neuropathy, SDSD, standard deviation of successive differences; eNOS, endothelial nitric oxide synthase; METs, metabolic equivalents for physical activity; VO_2peak_, peak oxygen uptake; SDANN, the standard deviation of the 5 min average NN intervals; TS, turbulence slope; VLF, the very low frequency; 30 s STS, sit-to-stand in 30 s.

## Data Availability

Not applicable.

## Supplementary Materials

The following supporting information can be downloaded at https://www.mdpi.com/article/10.3390/healthcare11192668/s1, PRISMA 2020 checklist.
